# New policies, old habits: why and how Dutch mental healthcare moves towards collaboration

**DOI:** 10.1186/s13033-026-00708-x

**Published:** 2026-05-18

**Authors:** T.S. van Dijk, C. de Koning, M. Felder, W.K. van der Scheer, R.T.J.M. Janssen

**Affiliations:** 1 Erasmus School of Health Policy & Management, Erasmus University Rotterdam, Burgemeester Oudlaan 50, Rotterdam, 3062 PA The Netherlands; 2 GGZ inGeest, Overschiestraat 57, Amsterdam, 1062 HN The Netherlands; 3 Tranzo, Tilburg School of Social and Behavioral Sciences, Tilburg University, Prof. Cobbenhahenlaan 125, 5037 DB Tilburg, The Netherlands

**Keywords:** Mental healthcare, Health insurers, Policy reform, Collaboration, Institutional logics

## Abstract

**Background:**

Hindered by staff shortages, high workloads and rising waiting lists, mental healthcare services across Europe struggle to provide accessible, appropriate, and timely care for those who need it. In the past, governments tried to tackle large-scale issues in mental healthcare with profound policy changes, such as deinstitutionalization, decentralization and marketization. In the Netherlands, where mental healthcare is also under pressure, many policymakers and other influential stakeholders believe that policy changes aimed at stimulating collaboration between providers, third-party payers and other domains are a solution to the increasing challenges. This research analyses how a collaborative logic unfolds in a complex sector dominated by a market logic.

**Methods:**

By analyzing policy documents and conducting interviews with representatives of healthcare organizations and health insurers, we explored both the systssemic and cultural barriers that challenge collaboration in mental healthcare.

**Results:**

Findings show that, while collaboration is framed as a key strategy to resolve issues in mental healthcare, the entrenched market logic has fostered negative perceptions and lasting mistrust between mental healthcare stakeholders. These perceptions make collaboration difficult.

**Conclusion:**

Challenges related to collaboration are not only systemic or financial in nature but are deeply rooted in belief systems, norms and values. A fundamental shift in these areas is essential to foster a more cooperative practice in mental healthcare.

## Introduction

Hindered by staff shortages, high workloads and rising waiting lists, mental healthcare services across Europe struggle to provide accessible, appropriate, and timely care for those who need it [36, 49]. The recent COVID-19 pandemic only has further magnified these issues, as especially the mental wellbeing of young people and vulnerable groups experienced a negative impact [32, 45]. Overcoming such issues and improving mental wellbeing is a complicated task, that mental healthcare organizations and governments cannot overcome on their own [32, 62]. There is good reason why Hargrove and Glidewell [18] describe the organization of mental healthcare as one of the “impossible jobs” in public management. Mental healthcare deals with limited public support, low professional authority and societal stigmas. Furthermore, mental health issues are not easily resolved and require long-term commitment, making health outcomes diffuse. Moreover, professionals, scientists and policymakers often have different and disputed views on what good mental healthcare is [18].

To make it even more complex, in past decades, mental healthcare has undergone many policy reforms. These reforms reflect governments’ efforts to address profound issues that hinder access, quality and affordability of mental healthcare, but they often further complicate the organization of care. For example, “deinstitutionalization” – a process in which care is moved from institutions and hospitals to community-based settings – had to improve the recovery of patients in stimulating and less-isolated environments, while at the same time saving money [31, 35, 38, 42]. “Decentralization”, subsequently, shifted elements of mental healthcare to primary care or placed it under the responsibility of local governments. The goal was to ensure that patients received care close to their home and better tailored to their needs [16]; And in some countries, “market-oriented reforms” to improve the efficiency and affordability of care were implemented [5, 33, 61].

In the Netherlands, mental healthcare has faced each of these policy changes; deinstitutionalization took off in the early 2000s, elements of the market were implemented in the early ‘10s and decentralization from government to municipalities took place in 2015 [61]. These transformations led to a new level-playing field and changing roles for healthcare providers, patients and purchasers of care (e.g., municipalities and health insurers). As part of the deinstitutionalization, healthcare providers had to reduce their number of “beds”, while also trying to sustain a solid business operation. New purchasers of care were introduced in a context of managed competition and decentralization. Healthcare providers now negotiate with health insurers, municipalities and regional procurement offices and are competing with other providers of care [61]. The rate at which these policies were implemented, and the fundamental rearrangement of responsibilities and financial risks resulted in a complex and fragmented system for all [9]. In addition, the financial crisis of 2007 led to new financial regulations for banks and health insurers, making it difficult for mental healthcare organizations to access financial resources needed to facilitate required changes [54].

In recent years, criticism of how mental healthcare is organized has resurfaced, as staff shortages and waiting lists continue to grow while patients experience a lack of cooperation and uniformity between specialized and basic mental healthcare ([19]; [5]). The urgency to change policy direction is rising once again, and many see collaboration and a more networked mental healthcare sector as the answer to address systemic [43] or “wicked” problems [13, 23], while simultaneously improving healthcare quality, coordinating healthcare services, and reducing costs [17, 25, 56]. To do so, policymakers and field parties have increasingly been supporting and engaging in initiatives that promote collaboration between mental healthcare providers, purchasers and other related health domains (e.g., *Integral Health Accord; Healthy and Active Life Accord* in the Netherlands; [34, 43]). This move towards a collaborative steering mechanism, however, conflicts with existing policy arrangements that emphasize competition and decentralization [41]. Like any other policy shift, moving from competition to collaboration is accompanied by challenges and friction, as it brings underlying values and practices into conflict [44]. In mental healthcare, these tensions have made it particularly difficult to implement and sustain collaborative principles, with obstacles such as power struggles, trust issues and a lack of coordination [2, 30].

This research will focusses on the past couple of years (2019–2024) during which system parties (e.g., policymakers, health insurers and healthcare providers) have been promoting new organizational principles in the Dutch mental healthcare setting while continuing to operate within a market-oriented context. While extensive research has examined pro-competitive reforms and their effects on the broader Dutch healthcare system [28, 55, 61], studies specifically focusing on how policy language and practice interplay during shifts towards collaboration within mental health remain scarce, especially in exploring how these dynamics unfold across different system actors. Consequently, our study addresses this gap by asking: *How can collaboration solve current issues in mental healthcare*,* according to system parties and providers*,* and what challenges do they face in balancing collaboration and competition in practice?* We will show how collaboration is presented in policy language and argued for in policy language. We then provide insights into how collaboration takes place in practice and how different perspectives and perceptions of parties collide. For this study, the focus is on integrated mental healthcare organizations and care services financed by health insurers under the Health Insurance Act (curative care lasting less than three years). We narrow our focus to this segment because it has been subject to all prior reforms, including market-oriented reforms, making it a strategic site for analyzing the evolving narrative from competition toward collaboration.

In the following paragraphs, we first provide a more extensive description of Dutch mental healthcare, how it is organized and how policy changes over the course of several years have fragmented the sector. Thereafter, our theoretical framework zooms-in on the underlying values of two different institutional logics – collaboration and competition – that meet in contemporary policy strategies and practices to overcome challenges in Dutch mental healthcare. The method section explains our approach and analysis, followed by the result section, and we conclude with a discussion.

## Re-organizing dutch mental healthcare

The organization of the Dutch mental healthcare sector has been subjected to many policy reforms over the past decade. Up until 2008, all mental healthcare services were covered by the Exceptional Medical Expenses Act (EMEA) and tendered by regional procurement offices. This drastically changed when principles of managed competition were adopted in Dutch healthcare and implemented in the mental healthcare sector from 2008 onwards. While most mental healthcare services remained under the EMEA until 2014, curative mental healthcare (consisting of services with the duration of up to one year) was added to the Health Insurance Act (HIA). As a result, health insurers became responsible for purchasing curative mental healthcare. Prices and quantities of mental healthcare services were no longer fixed, but annually negotiable. A new funding system based on Diagnose Related Groups (DRG’s) was introduced for these contracts. Initially, the health insurer with the highest market share in a region acted as representing health insurer. This arrangement was however abolished in 2014, meaning an increased burden for providers to negotiate with multiple health insurers instead of only the representative [61]. As part of the pro-competitive reform, mental healthcare organizations became furthermore responsible for the building and maintaining of their real-estate and the financial stability of their organization. As a result, integrated mental healthcare organizations often extended their area of operation or merged with others [21].

In 2014, curative mental healthcare underwent further restructuring into two segments. General practitioners’ practices were tasked with treating patients with mild mental health problems and referring patients with moderate conditions to the ‘generalist basic mental health segment’. Patients with severe conditions were helped within the ‘specialized mental health segment’. (Westra et al., [61]; [58]). A year later, the sector was further fragmented when services within curative mental healthcare were extended to the duration of up to three years. In that same year, child and youth mental healthcare, protected living, day care and ambulatory coaching were decentralized to municipalities. Furthermore, the Long-term Care Act was introduced. This law covered mental healthcare services with the duration from three year onwards and remained the responsibility of regional procurement offices [22, 61]. With these final changes, the former EMEA was completely dismantled, transferred, and decentralized.

For integrated mental healthcare organizations that provide different types of services, the sector became increasingly complex. In a short period of time, they had to change their way of thinking and doing to align with the principles of managed competition and faced increased financial risks. They furthermore had to adapt to different reforms and purchasing parties (i.e., municipalities, health insurers, regional procurement offices and in some cases the Ministry of Justice for forensic care) with each their own contracting principles (e.g., annual negotiations and public tenders). On top of that, the Ministry of Health and field parties had in 2012, decided to reduce the number of beds by 30% to further accelerate deinstitutionalization. All in all, the sector was presented with a huge financial and operational challenge [21].

## Competition and collaboration as institutional logics

The accumulation of policy reforms not only reshaped the organization of Dutch mental healthcare and redistributed responsibilities among stakeholders, it also resulted in the coexistence of different institutional logics within the sector [11, 40, 47]. Institutional logics refer to a belief system with coherent norms, rules and values that shape and are shaped by the behavior of actors. Logics are embedded in cultural and societal contexts and shape organizational practices and decision-making [15, 48]. Different institutional logics (in mental healthcare) constantly interact, collide and are continuously being re-negotiated, thereby changing and influencing one another [1, 44]. Institutional logics also shape policies (and vice versa), and over time, different dominant logics have influenced the introduction of new policies in the mental healthcare sector. For example, the idea that market incentives could increase efficiency in mental healthcare have been an important driver for market-oriented policy reform, as well as the idea that mental healthcare should be organized within the lived environment of patients rather than in large-scale and isolated institutions. Since these policies accumulate, rather than being replaced, mental healthcare has become a fragmented and layered sector in terms of governance, financing and operational regimes [12, 29, 50].

In current discussions on how to organize mental healthcare, two underlying institutional logics emerge, which are the focus of this study: a market logic and a collaborative logic. To better understand the rules, values and assumptions of these logics, we will describe them as ideal typical institutional logics. These ideal typical descriptions serve as a framework to help guide our analysis but should not be viewed as a description of actual practices, which are complex, involve a myriad of logics, and may change in dominance over time.

Within an ideal-typical market logic, organizations are seen as competitors that try to optimize services and maximize profits. To do so, they need to work efficiently and innovate. Patients, or consumers, are perceived as rational actors who can make their own trade-offs and choose the most desired provider. Both providers, costumers and (third-party) buyers are associated with opportunistic behavior, follow private interests and their relation is a transactional one in which goods and services are exchanged. Healthcare is further seen as a commodity in which clients have the freedom to choose which care they want to receive, from who and when. In a perfect healthcare market, it is believed that an equilibrium, where supply and demand for care are balanced, will be reached. Underlying values in a market logic are rationality, resourcefulness and consumer centricity [3, 39, 51].

In an ideal-typical collaborative logic, different actors (both public and private) work together to achieve a common goal. They often share the same values and ethics or reach common ground through democratic decision-making, dialogue and deliberation. Communication and mutual coordination in these, often informal, organizational forms are therefore important. Actors are supposed to behave beyond self-interest, are dependent on one another, share responsibilities and co-produce their strategies. It is key to align different stakes, responsibilities and resources within a network. Important values are trust, reciprocity and mutuality [4, 6, 10].

Both a market logic and collaborative logic are present within mental healthcare organizations. They continue to operate within a context that has long been governed by a market logic, and their thinking and behavior have been heavily influenced by it. At the same time, emphasis on collaboration and organizing care in networks is becoming increasingly dominant. Efforts to collaborate can thus be fraught with value conflict, expressed in different ideas about what problem to solve, how to solve it and how to be accountable [60]. Schuurmans et al., [41], for example, show that market principles incentivize actors to prioritize the interests of their own organization above solving collective problems such as staff shortages and fragmentation in healthcare. How parties in mental healthcare overcome struggles in implementing collaborative ways of working in a market-oriented context is the focus of this research. Norms, values and behavior are not easily twitched or re-shaped. They are reproduced in every-day, mundane activities and expressed in language and symbols. They inform how actors think and behave towards each other and within their institutional context. This research, therefore, takes notion of the intentions that new policies embody and pays attention to the challenges that take place in practice.

## Methods

### Data collection

This study is based on a narrative document analysis and semi-structured interviews. For the document analysis, inclusion criteria were (1) publication between 2019 and 2024, (2) authorship by dominant system parties in the Dutch mental healthcare system, (3) a focus on the entirety of the mental healthcare sector and (4) a focus on the problems of mental healthcare and possible and forward-looking solutions for this. Excluded documents reflected negotiated or consensus-based positions between relevant stakeholders (e.g., *Integral Health Accord* and *Healthy and Active Life Accord*), documents limited to a narrow theme (e.g., prevention or financing) without offering a sector-wide perspective, and parliamentary debates or political discussion, which emphasize short-term political issues rather than strategic visions. This resulted in three policy documents (see Table 1).

Publishers of the selected policy documents are the Association for Dutch Health Insurers (*Zorgverzekeraars Nederland)*, the Association for Dutch Mental Healthcare Providers (*De Nederlandse GGZ*) and the Ministry of Health. The first is an association that represents all ten health insurance concerns in the Netherlands and promotes their interests. The organization is a key player in the Dutch policy arena and frequently publishes memoranda, position papers and guidelines. The Association for Dutch Mental Healthcare Providers represent the interests of both mental healthcare organizations and their professionals. They provide a platform for these organizations and professionals to meet and exchange knowledge. The association is politically involved and often advises or tries to steer political and societal debates. By publishing policy related documents, these associations directly express their wishes and try to influence government, policymakers and other parties in the field. In Dutch healthcare, it is common that umbrella and representative organizations or care associations are involved in policymaking to build consensus and gain legitimacy [26]. The Ministry of Health is end-responsible for Dutch healthcare and another important policymaker regarding mental healthcare. The Ministry increasingly pays attention to mental health issues among youth and is keen to put an end to long waiting lists and thus, guarantee access to care. At the time of publication of the first and second document, elections were imminent. The third document was published shortly after the elections.

**Table 1:** Policy documents

Title		Publisher	Date	Type of document
**Document 1**	Mental healthcare in 2025.The future of mental healthcare	Association for dutch health insurers	17/7/2020	Position paper
**Document 2**	Mental healthcare: tailor-made and on time	Association for Dutch Mental Healthcare Providers	1/5/2020	Input for election program
**Document 3**	The landscape of mental healthcare	Ministry of Health	19/5/2021	Discussion paper

The document *Mental healthcare in 2025: The future of mental healthcare* [7] outlines objectives for the future of mental healthcare on nine themes: supply, collaboration, demand, quality, governance, personnel, society, financing and regulation. The second document, *Mental healthcare: tailor-made and on time *[8], provides input for the elections and targets mainly government. They discuss “rearrangement of care”, “trust in professionals”, “prevention and early detection” and “societal challenges”. The final document, *the landscape of mental healthcare* [59], is an inventory of possible solutions for problems in the mental healthcare sector. It provides options for “prevention and organizing care around human beings”, “organization and autonomy” and “innovation and work pleasure”. The Association for Dutch Health Insurers and the Ministry of Health further indicate that they consulted clients, experts, professionals and other parties for their document. Nevertheless, the presented vision is their own. The three documents provide a good understanding of how involved parties at the national level (regulator, financer and provider) view the mental healthcare sector, their key problems and possible solutions.

In addition to the policy analysis, we held fourteen interviews between February and March 2021 with representatives of mental healthcare organizations and health insurers. This provided us a practice-oriented view on the mental healthcare sector and the opportunity to compare narratives of “policy ideas” with practices and perspectives. Thereby, being able to unravel how the problems and developments in the mental healthcare sector discussed in policy documents are being experienced and framed locally and in practice.

Mental healthcare organizations were represented by their executives. We specifically narrowed our focus to integrated healthcare organizations since they have been subjected to all previous reforms and are seen as the backbone of mental healthcare. There are twenty-seven integral mental healthcare organizations in the Netherlands. We endeavored to interview organizations with different sizes and operating in different regions. Since the group of executives is often very busy, especially in COVID-19 times, we first contacted those that were accessible through the network of author two and four. Other executives were often referred to by their colleague-executives.

The representatives of health insurers worked as policy advisors and (managing) purchasers. They are actively involved in maintaining relations with mental healthcare organizations, developments in the sector and strategic policies. With ten health insurance concerns active in the Netherlands, we interviewed seven and covered both those with a larger market share and those with a smaller. The second author contacted and interviewed 15 respondents (see Table 2) and covered the following topics: the relation between mental healthcare organizations and health insurers, inter-organizational collaboration, and their vision on recently published policy documents and on possible solutions for the current problems in the sector (see appendix 1 for the interview guide). Due to covid restrictions, all interviews were held online. Data collection continued until no new themes emerged in the interviews, suggesting that data saturation was reached and that the range of perspectives captured was sufficient to address our research aims.

**Table 2:** Respondents

Function respondent	Organization	N
CEO	Integrated mental healthcare organization	4
CFO Member of the Board	Integrated mental healthcare organization Integrated mental healthcare organization	1 1
Head of department	Psychiatric department hospital	1
Advisor mental healthcare sector	Health insurer	5
Manager purchasing department	Health insurer	1
Account manager	Health insurer	2
**Total**		**15**

### Data analysis

Our analysis followed an abductive thematic approach [46]. We iteratively moved between the empirical material and theoretical lenses in order to develop an interpretation that addressed our research question. This allowed new insights to emerge directly form the text, while also enabling us to understand the data through conceptual perspectives, of which the framework of a collaborative and competitive logic provided the most analytical value. Through this iterative engagement with data and theory, we refined our codes and gradually build broader themes (e.g., conflicting interests, fragmentation, trust, transparency, coordination, alignment and shared responsibility). A set of codes was developed and continuously updated throughout the analysis to ensure consistency across sources.

We analyzed three policy documents. These were first coded close to the text to capture emergent topics. We then compared the codes to identify common and divergent themes across the documents. Our focus was on how problems and solutions for mental healthcare were presented: we paid attention to language use, the authors’ position representation, their involvement and interests in policymaking, the ways problems were defined, and the solutions that were proposed. We also examined what remained implicit or unsaid in the documents.

The interviews with executives and health insurers were audio-recorded, transcribed verbatim and thematically coded in Atlas.ti. The interview guide contained questions on several topics. Respondents were asked about their relationship with health insurers or (other) mental healthcare providers. They were asked how these relationships were shaped in terms of trust, power, legitimacy, and influence in organizational decision-making and in processes of contracting and negotiation [53]. Further questions addressed experiences with collaboration initiatives and how collaboration might serve as a solution to systemic challenges in the sector, including long waiting lists, the treatment of complex patients and staff shortages. Finally, respondents were asked how, in their view, current problems in mental healthcare should be addressed in their opinion, and what their vision was for the future organization of the sector.

The first and second author both coded the interviews independently, compared their coding, and refined the codes in iterative rounds. Codes from both the documents and the interviews were then structured into thematic clusters and connected to one another, resulting in a broader picture of how collaboration is experienced in practice and how health insurers and healthcare executives frame one another. In additional meetings with all authors, the thematic structure and its implications were discussed, challenged, and further developed.

## Results

The result section is divided into three parts. The first demonstrates the “why” of collaboration. An analysis of the selected policy documents reveals to what end parties in healthcare believe that we should make the move towards a collaborative governance regime. The second part combines an analysis of the policy documents and interviews to show how parties experience cooperation in practice. Here, we further elaborate on the elements that are pointed out in both the documents and by our respondents as obstructing collaboration. While policymakers attempt to propose solutions, practices often prove challenging due to deeply entrenched opposing perceptions of “the other”. These perceptions are further elaborated upon in part three.

### Part 1: collaboration as holy grail

The authors of the three policy documents – the Association for Dutch Health Insurers, Association for Dutch Mental Healthcare Providers and the Ministry of Health – all start from the premise that mental healthcare is under pressure. An increased amount of people call upon mental healthcare services and for many of them, care is not easily accessible due to long waiting lists and the unavailability of suitable care. There are also staff shortages, financial issues and continuous policy reforms that increase work pressure and administrative burdens. These issues are acknowledged in all documents, although the framing differs in line with everyone’s specific role. The Association for Dutch Health Insurers emphasizes the disbalance between demand and supply of mental healthcare service [7; p. 6], while the Association for Dutch Mental Healthcare Providers approaches the problem from the perspective of clients, family members and professionals. They identify the suffering experienced by clients and families, as well as the loss of enthusiasm among professionals for their jobs [8; p. 5] . The Ministry of Health takes a policy perspective and aims to ensure public values such as quality, accessibility, and affordability of care [59; pp 3-5].

To solve these issues, the documents describe specific solutions. All of them require close collaboration between different stakeholders in the sector and are thus a (sometimes inexplicit) call for closer cooperation. *Mental healthcare in 2025*, for example, mentions that every stakeholder in the sector must take on a certain responsibility and solve the above-mentioned problems jointly. It states: “we must realize this shared belief together” [7; p. 7]. In *Mental healthcare: tailor-made and on time*, the authors mention that “if mental healthcare and societal partners work closer together, everyone who needs it, will receive care and support that is tailor-made and on time” [8; p. 3]. The ministry is also set on collaboration: “Instead [of growth], the mental healthcare sector must search for collaboration with other relevant domains (such as housing, participation/work and youth care) […]” [59; p. 7].

Collaboration would solve three problems. First, organizing care in collaboration with different providers, on a local or regional level and around the patient is supposed to tackle a lack of adequate and suitable care and waiting lists. The Association for Dutch Mental Healthcare Providers pleas for new organizational arrangements of care on a national, regional and local level. Care on the national level should entail specialized centers for complex problems, while acute, crisis and addiction care are organized on a regional level. Collaboration should especially take place on the local level between mental healthcare providers, the social domain, GP’s and societal partners such as housing corporations and (debt) counselling. Organizing care around the patients and in the region is also an important cornerstone in the policy document of the Ministry of Health. They assume that on a regional level, more knowledge regarding patients and suitable care is available; and that it provides professionals with more autonomy to act. Organizing care in this way, requires a network of professionals from different care domains and a close coordination of tasks. The Ministry makes itself responsible for creating the right circumstances to make this happen.

"The government takes the lead in developments in the mental healthcare sector, amongst other actions, by setting stringent conditions for the development of necessary rules and regulations. In cases where developments are delayed, the government intervenes. Additionally, the government fosters greater collaboration across domains and stimulates the development and implementation of (new) collaboration models.” [59; p. 15]

The Association for Dutch Health Insurers adds that collaboration should not only take place between professionals but also on a governance level, between healthcare organizations and with health insurers.

The second issue that all three documents discuss, is the lack of focus on prevention. Policymakers believe that by stimulating prevention and reframing societal views on mental health insurers, the need for mental healthcare services will reduce. However, to achieve prevention and establish new views on the use of mental healthcare, the effort of many different stakeholders is necessary. They must coordinate their actions and work together to achieve prevention. To finance such efforts, policymakers propose combining the financial budgets of health insurers and municipalities, with the latter acting as the main coordinators. GP’s and professionals in the social and care domain can do early detection and provide treatment together.

The third pressing issue that the mental healthcare sector contends with is staff shortages. The writers of the policy documents propose that collaboration can be part of the solution for this problem. To lower work pressure for professionals, the Association for Health Insurers suggests to better adjust activities between organizations. This requires coordination, increased contact moments between providers and thus collaboration. The ministry proposes regional learning networks as a solution for staff shortages. These networks can be established for self-employed professions, pensioners and others involved to join with the goal to support professionals during night and weekend shifts and supervise employees working in mental healthcare.

Thus, the policy ideals of the ministry and associations are congruent; all three argue that the multitude of problems in the sector can only be solved when parties work together. Characteristics of a collaborative logic are clearly present in the documents, such as the formation of networks with equal parties and the goal to solve shared problems. However, policymakers never become very concrete on how to establish and maintain networks of collaboration [52] nor what regulatory or operational changes are required. Instead, they propose a vision and ideal situation, presuming that collaboration can solve all systemic problems. What misses, for example, is how the rules, regulations and culture that have been established under a market logic relate to collaboration initiatives or what consequences this has for newly established practices. We, therefore, make the step towards the second and third part of the result section in which we zoom-in on practices. More specifically, we display the collaborative practices between healthcare providers in the sector and the existing perceptions between health insurers and healthcare providers. With this, we illustrate the (mis)alignment of current practices with new policy narratives.

### Part 2: collaboration in practice

The interviews show that executives, representing their mental healthcare organization, and health insurers also see collaboration as a solution for the pressing issues the sector is facing. They share the same intention, as laid out by policymakers, to cooperate and stimulate partnerships in order to deal with urgent issues: staff shortages, higher demands for care and waiting lists. They believe that by learning from one another and expanding their knowledge, collaboration proves a better ground to solve such issues. Executives and health insurers explain that most of the current initiatives that have been set-up take place around a specific topic, such as quality improvement, reducing waiting lists or are driven by knowledge-sharing. In line with a collaborative logic, some healthcare parties have come together and work together towards a certain goal. The quote below illustrates that collaboration also necessitates a shift in perspective, viewing the other party not as a competitor, but as a partner.

“There are increasingly more organizations that share and exchange knowledge. That is much improved compared to ten years ago. These organizations don’t view the other as competitor” *Executive integrated mental healthcare organization*.

So, for some mental healthcare organizations the pressing situation in the mental healthcare sector leads to close collaboration. One of the respondents, for example, explains how their organization together with others have combined their purchasing practices and information systems and have a joint approach regarding HR and personnel related topics to deal with staff shortages. Another executive explains how a collaboration initiative changed the unstable relation between their organization and health insurers and municipalities for the better and strengthened mutual understanding of each other.

“With our substantive and innovative projects […] health insurers are closer connected to us. We feel seen.” *Executive integrated mental healthcare organization*.

Despite these examples, for many others it is difficult to put ideas of collaboration into practice. Respondents point to several obstacles. Healthcare executives mention that collaboration is often obstructed by current laws and regulations stemming from a market logic (e.g., competition law), and further complicated by the multiplicity of collaboration initiatives and the relation-management this requires. Healthcare executives have no clear overview of all collaboration initiatives, while they are required to steer and organize them. Another returning difficulty are the dispersed financing systems and different purchasing parties within the mental healthcare sector (i.e., health insurers, municipalities, regional procurement offices and the Ministry of Justice). Respondents point to how financial systems are legally demarcated and not equipped to facilitate care across domains. With inter-sectoral collaboration, the question then becomes which party (e.g., health insurer, regional procurement office and/or municipality) is going to finance collaboration and for how much?

“The current system is not equipped to create the right conditions for collaboration, for collective action. It would help if all health insurers said: “Here is a large pot of money for a certain region to tackle a certain condition. Show us a joint proposition on how to achieve a breakthrough.” But now it is fragmented to different organizations, health insurers, you name it. There are a million care providers. Currently, we are dealing with twenty different partnerships and contacts. That does not work.” *Executive integrated mental healthcare organization*.

The many different opinions and experiences with collaboration show that parties find it difficult to decide where to start and how to overcome obstacles along the way. For some, small initiatives (e.g., collaboration around specific issues and through knowledge-sharing) help grow trust and good relations between involved parties. For others, it is difficult to set-up collaboration initiatives as they miss the appropriate preconditions (e.g., suitable regulation and financing mechanisms). In the next section, we dive deeper into what is behind the mentioned obstacles and why it is so difficult to overcome them when all parties are convinced of the importance of collaboration. We therefore look at the ingrained perceptions that parties have of each other and how that informs their actions.

### Part 3: untangling existing perceptions

The interviews show that most parties feel hindered by a (recent) past in which cooperation between healthcare organizations and financed by health insurers was not desired, nor possible. In a system of managed competition, cooperation is seen as an undesired pooling of power. Besides, many interviewed executives refer to a past in which health insurers would not be able to finance far-reaching collaborative initiatives without permission of the Market Authority and where relations between health insurers and healthcare organizations were full of tension. The actions of insurers were even perceived as unreasonable by healthcare organizations and negotiations as unnecessary harsh (see also, [27, 53]). Insurers, in turn, speak in similar distrusting ways of healthcare organizations.

“No matter how hard we all try, there is still a lot of distrust. We [health insurers and healthcare organizations] have our own agenda. There are shared interests and a lot of opposite interests. Especially when things get a bit tense, which is often towards the end of the year when negotiations for the new year start again. Then you see that the interests really differ, there is a lot of distrust and insurers often react by incorporating incentives into the contracts that limit risks as much as possible.” *Advisor mental healthcare sector for health insurer*.

Also, when it concerns attempts to collaborate, distrust remains an important obstacle between parties. One of the health insurers explained, for example, that when they organized sessions with providers in a specific region to exchange information on good practices and failures, some organizations were afraid this would have consequences for their contract with health insurers.

The troubled relation between mental healthcare providers and health insurers stems from their underlying perceptions and frames they have of each other. Healthcare executives experience health insurers as financially driven organizations with little substantial knowledge of the sector. Annual negotiations are often perceived as difficult and a fight and when negotiations get tough, health insurers stick to rules and fixed patterns. According to executives, health insurers want control and think that change within healthcare organizations is easily accomplished.

“The health insurer told me they wanted to purchase less care from us. I told them: “well, that is strange, because not so long ago, you declared us as the best provider of addiction care in the Netherlands. Why do you now want to purchase less? I mean, if you can show that I perform less in price and effectivity compared to others, then it is fine, then we can talk.” But then they remain silent. I think insurers did not really invest in transparent purchasing practices.” *Executive integrated mental healthcare organization*.

Health insurers on the other hand, feel that healthcare providers specifically want to increase services and prices but that a clear justification and the data to back it up often misses. Health insurers experience a lack of transparency from executives and find that mental healthcare organizations miss sound operational management and clearly organized integral processes. They perceive larger integral organizations to have a lot of power, determine the field and miss an incentive to innovate or change.

“Then we are told by healthcare organizations: “you have been paying far too little for years, we need 10% extra.” Then we ask: “Why? Where is the substantiation for that? Can you demonstrate financially that it is indeed necessary? Can you demonstrate that you are doing everything to manage costs?” I miss that with many healthcare organizations. […]. If a healthcare organization thinks: “I should add an extra 10% in my negotiations, so that I might end up with 4% extra, then it is no longer about care or business operations, but it becomes trade, a bit of arm-wrestling, who is the strongest?” *Account manager health insurer*.

Respondents, however, also indicate an interesting deviation from these dispositions. Most Dutch health insurers have a specific region in the Netherlands in which they have the largest market share. Within these regions, health insurers and healthcare organizations frame each other more positive and feel closer connected. Here, health insurers experience more financial and operational transparency from healthcare organizations and a willingness to cooperate and change. Healthcare executives find their health insurer more involved and interested in quality of care and improving healthcare processes. They describe them as open and willing to think along. The reason why health insurers limit their focus to their own region is one that is very much prompted by a business-like mindset:

“It is important to us that such an organization is from a region where we have a relatively high market share. A close collaboration with organization X, where we have a 15% share, is not that interesting to us, because we also provide capital investments. And then we invest in the territory of another health insurer.” *Advisor mental healthcare sector for health insurer*.

In these cases, the dependency between the dominant health insurer and dominant provider makes it easier to realize cooperation and good relation management in a region. Experiences, in short, differ from region to region, and from relation to relation. We observe that for executives and health insurers, their own specific experiences are leading in whether they feel collaboration as a promising solution. Executives are often positive regarding the largest health insurers in their region, and negative towards others. For health insurers the same applies. The ingrained perceptions of suspicion and distrust and the actions that follow stem from a time that a market logic was dominant in mental healthcare. Health insurers and healthcare organizations are currently trying to combine two different regimes: they need to negotiate over contracts, while at the same time cooperate and find agreement over substantive themes in the mental healthcare sector. This results in relations between healthcare organizations and health insurers that bounce back and forth between trust and distrust. Much depends on acquired trust, being able to make decisions beyond self-interest, and the willingness to cooperate while the right preconditions might not yet be in place.

## Discussion and conclusion

In many European countries, mental healthcare is struggling with growing staff shortages, high workloads and increasing waiting lists. The sector is at the same time fragmented and difficult to organize, because of its contested nature [18] and consecutive policy reforms [61]. The urgent problems embedded within complex mental health systems require coherent and well-aligned solutions. Some countries facing similar challenges have pursued community-based and integrated care approaches, which rely on collaboration across multiple intersectoral domains and actors [14, 20, 24, 34]. Others have focused on formal waiting time standards and stepped-care models to triage demand and improve timely [37]. In the Netherlands many policymakers, providers and purchasing parties consider collaborative strategies to be the most promising path forward. In recent policy documents collaboration between stakeholders within mental healthcare is presented as holy grail: expected to solve a lack of suitable care, waiting lists, staff shortage, stimulate prevention and reduce the need for mental healthcare. The representatives of healthcare organizations and health insurers we interviewed share this belief in a collaborative logic, but often feel obstructed by rules, regulations and financial systems that stem from a time in which a market logic prevailed. While the policy arena seeks to combine present market-like logics with that of collaboration, this proves difficult in practice. Since the implementation of a market logic, relations between providers and health insurers deteriorated and were based on distrust [53]. For more than a decade, healthcare providers and purchasers were supposed to work along principles of managed competition, which emphasized competition and entrepreneurship. It inherently led to distrust among healthcare providers that perceive the other as competitor and between mental healthcare organizations and health insures that negotiate over contracts. The move towards collaboration requires a shift in attitude, one in which transparency, shared interests and common purpose can grow. While we do see that collaboration between parties in mental healthcare develops, actors remain stuck in a liminal (in-between) space between competition and cooperation and struggle to move forward and find a new way of working together – in light of their past. While rules, regulations and financial systems are cited as obstacles to collaboration initiatives, we found that the persistent and entrenched perceptions that stakeholders hold of each other also significantly contribute to deteriorating trust and hinder collaboration.

Using an institutional logic approach helped dissect ideas about how to organize mental healthcare as both a market and as collaborative network. While different logics can co-exist, they often lead to conflict, value tension and an institutionally layered system [12, 50]. For example, a market logic fosters actors to follow their own interests instead of setting these aside for the greater good [41]. Our research adds to this body of knowledge by showing that it is a market logic that led to distrust, negative perceptions and suspicion of other stakeholders, making it difficult to collaborate. Challenges regarding collaboration are therefore not only systemic or financial issues, but require a shift in belief systems, norms and values, before practice can follow. A move towards more collaboration will thus require an “institutional” shift in the internalized modus operandi of these parties.

The researched policy documents do not consider any of these intricate complexities of reality, nor do they recognize the embedded roles and frames and underlaying norms, values and relations of involved actors. The outcomes thus show how ideals among policymakers on a national level, need to be made “fit” for regional and local practices. Policy and practices need to be actively aligned, and executives could play an important role in this, as boundary workers [52]. However, our research also shows that executives first need to deal with their own past experiences before they are ready to take on that intermediary role. They themselves have to make the change from distrust to trust, from old to new ways of perceiving and dealing with health insurers, municipalities and other involved parties. Building and maintaining trust requires constant work, lots of deliberations, patience to take steps forward and backwards, searching mutual goals and to be open about expectations [57].

Managing mental healthcare does really seem like an impossible job considering the challenges and complex institutional context of the sector [18]. Whether collaboration between different stakeholders in mental health will take off, if it can remedy the extensively discussed problems and whether that will result in better care for patients remains to be seen. For now, the challenge for all actors in mental healthcare seems immense and sharing this responsibility might offer some relief. Crucial is that the already fragile trust between parties is not harmed and can grow. The discussed policy documents leave a lot to the imagination, offering opportunities for healthcare organizations, health insurers, municipalities, social domain and other relevant stakeholders to shape new initiatives and structure their own governance. The gap that currently exists between policy aspirations and the complex realities of practice can serve a functional purpose: it highlights the problems and obstacles to collaboration, making visible where interventions are needed.

### Strengths and limitations

This study provides in-depth insights from key policymakers in the Netherlands, highlighting their vision for the future organization of mental healthcare, while also examining how these policy ideas interact and sometimes conflict in practice. By combining interviews with analysis of policy documents, we were able to triangulate perspectives, enhancing the credibility of our findings and offering nuanced understandings of the dynamics at play.

Several limitations should be noted. Stakeholders from the Ministry of Health were not included, which may have narrowed the policy perspective. Additionally, participants may have provided socially desirable responses, and as the study relied on interviews rather than direct observation, our insights into “practice” reflect perceptions rather than observed behaviours.

## Data Availability

The interview data generated and/or analyzed during this study are not shared as individual privacy could be compromised. All other data is available in the public domain.

## Appendix 1. interview guide

*Relationship between mental healthcare organizations and health insurers*

- How would you characterize your relationship with health insurers / mental healthcare organizations?
- How is this relationship shaped and organized within your organization?
- How does the contracting and negotiation process with health insurers / mental healthcare organizations proceed?
- Are there differences in the relationship with different health insurers / mental healthcare organizations? If so, what are those differences?
- How would you describe the quality of the relationship in terms of trust, accessibility, constructiveness, fairness, and productivity (and any other elements you consider relevant)? Can you provide concrete examples that illustrate these aspects (or a lack thereof)?
- Has your relationship with health insurers / mental healthcare organizations changed in recent years? If so, how and why?

*Inter-organizational collaboration*

- How would you characterize the relationship between mental healthcare organizations in the region?
- How would you describe the quality of the relationship in terms of trust, accessibility, constructiveness, fairness, and productivity (and any other elements you consider relevant)? Can you provide concrete examples that illustrate these aspects (or a lack thereof)?
- In light of the developed vision documents, how should the relationship ideally look? What is currently missing, and how can we get there?

*Vision on recently published policy documents and possible solutions for current sector problems*

- What problems do you see in the mental healthcare sector, and how could these best be addressed?
- Are you familiar with recently published policy documents, and/or did you participate in their development? Are they broadly supported and why (not)?
- What is your opinion of the policy documents, and why? Do they address the problems the sector is facing and why (not)?
- How large do you think the influence of these policy documents will be on the future of mental healthcare? Do they have consequences for negotiations and why (not)?
- Are the policy documents translated into the policies of health insurers / mental healthcare organizations? If so, how and why?
